# Time of Day and Sleep Deprivation Effects on Risky Decision Making

**DOI:** 10.3390/clockssleep6020020

**Published:** 2024-06-20

**Authors:** Noelia Ruiz-Herrera, Mia Friedman, Melissa A. St. Hilaire, Arturo Arrona-Palacios, Charles A. Czeisler, Jeanne F. Duffy

**Affiliations:** 1 Division of Sleep and Circadian Disorders, Department of Medicine, Brigham and Women’s Hospital, Boston, MA 02115, USA; nruizherrera@bwh.harvard.edu (N.R.-H.); msthilaire@bwh.harvard.edu (M.A.S.H.);; 2 Division of Sleep Medicine, Harvard Medical School, Boston, MA 02115, USA; 3 Department of Computer and Data Sciences, School of Engineering and Computational Sciences, Merrimack College, North Andover, MA 01845, USA

**Keywords:** decision making, BART, reaction time, constant routine, circadian rhythm, sleep deprivation

## Abstract

Previous research has revealed that daily variations in human neurobehavioral functions are driven in part by the endogenous circadian system. The objective of this study was to explore whether there exists a circadian influence on performance regarding a risky decision-making task and to determine whether the performance changes with sleep deprivation (SD). Thirteen participants underwent a 39 h constant routine (CR) protocol, during which they remained awake in constant conditions and performed the BART (balloon analogue risk task) every two hours. The mean pumps (gains) (*p* < 0.001) and balloons popped (losses) (*p* = 0.003) exhibited variation during the CR. The reaction time (RT) also showed significant variation across the CR (*p* < 0.001), with slower mean RTs in the morning hours following SD. A greater risk propensity was observed around midday before SD and a lower risk propensity after 29.5 h of being awake. The sensitivity to punishment varied during the CR, but did not follow a predictable trend. Further research using real monetary incentives and neurophysiological measures is warranted to elucidate these findings.

## 1. Introduction

Most neurobehavioral functions (including basic cognitive processes such as executive functions) show both sleep–wake homeostatic (related to the duration of time awake and effects of chronic sleep loss) and circadian (time of day) variations.

The effects of sleep loss on neurobehavioral functions are related to changes in brain networks, synapses, and even intrinsic neuronal membrane properties [1,2]. These effects can result in negative impacts on many aspects of cognitive performance [3,4,5,6,7], risks to safety due to inattention, and long-term risks to both medical and psychological health [8,9].

Although there is much evidence to support that sleep loss [5,10,11] and circadian disruption [12,13,14] impair alertness and vigilance, the effects of sleep loss and disrupted circadian rhythmicity on higher-order cognitive processing are less clear [15,16].

Previous studies concerning executive function, including supervisory control, problem solving, divergent thinking capacity, verbal creativity, flexibility, inhibition, cognitive set shifting, and risky decision making [17,18,19,20,21], have been carried out to explore whether the time of day or sleep loss impact performance. While some of those studies have reported significant impacts of the time of day on the performance regarding decision-making tasks [22,23], others have not [24,25], suggesting the need for additional studies to clarify whether the time of day impacts executive functions, and furthermore whether there is a circadian rhythm in performance on specific tasks of executive function.

In addition to studies of how executive function varies with the time of day, there have been studies on the impact of sleep deprivation (SD) on decision making, and these have shown inconsistencies. In a protocol of seven nights of sleep restriction followed by one night of acute SD (40 h awake), the participants took more financial risks during chronic sleep restriction but not following acute SD [26]. In another study where the Iowa Gambling Task was used, there was an increased risk in responses as the game progressed across 49.5 h awake [27]. The authors suggested that the change of strategy to more risky decisions after sleep loss was analogous to those seen in patients with lesions to the ventromedial prefrontal lobes. Similarly, in another study that kept participants awake for 24 h, the authors observed that choices involving a higher relative risk elicited greater activation in the right nucleus accumbens (an elevated expectation of higher reward) once the riskier choice was made. Concurrently, activation for losses in the insular and orbitofrontal cortices was reduced, denoting a diminished response to losses [28]. This same research group reported that 24 h of being awake evoked a strategy shift during risky decision making such that participants moved from defending against losses to seeking increased gains [29]. However, another study that used a multi-attribute decision-making task found no differences between sleep-deprived (33 h) and non-sleep-deprived subjects [30].

In summary, there appears to be no consensus on the effects of the time of day and sleep loss on decision-making tasks. However, study methodologies have differed markedly, and, to date, no data have been reported on risky decision making under controlled behavioral conditions across an acute sleep-loss protocol.

Therefore, the aim of this work was to explore whether there is a circadian rhythm in risky decision making, and, through the use of the constant routine (CR) protocol, to further explore how acute sleep loss impacts risky decision making in a sample of healthy young adults.

## 2. Results

Fifteen healthy adults (nine women, six men) between 21 and 31 years old (M = 26.00 ± 4.05 years) began a five-day inpatient circadian rhythm study. One male participant was disempaneled on day two due to COVID exposure, and one female participant was disempaneled on day four due to becoming ill. No data from either of those participants is included in the analysis presented.

Among the thirteen participants who completed the study (eight women, five men between 21 and 31 years old, M = 26.46 ± 4.16 years), there were occasional missing test sessions. One participant did not complete one session of the balloon analogue risk task (BART) during the baseline and another participant missed six of seven sessions of the psychomotor vigilance task (PVT) and five of seven sessions of BART during the baseline. One participant missed the second test session of the CR for both the BART and the PVT due to schedule delays. PVT data were not collected from one participant in session 10 of the CR and for one participant in session 1 of the CR.

Mixed model analysis with TIME AWAKE as a fixed factor and PARTICIPANT as a random factor showed that there was no significant effect of time awake on any BART or PVT variable across the seven baseline sessions (all *p*’s > 0.5). Mixed model analysis with the factors CONDITION (Baseline vs. CR), TIME AWAKE, and their interaction was used to compare the seven baseline day test sessions with the CR sessions. This found no significant effect of the condition (baseline day vs. CR) and no interaction (all *p*’s > 0.5), suggesting no learning effects on any of the BART or PVT variables.

**Figure 1:**
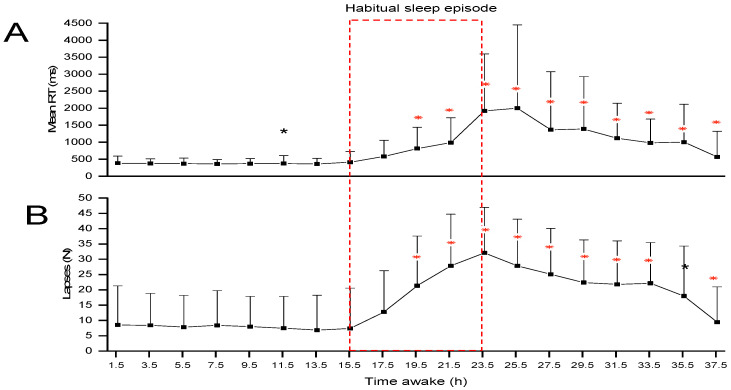
Mean (+standard error) reaction time (**A**) and lapses (**B**) on the psychomotor vigilance task (PVT) across the CR. The dashed red box indicates the timing of the habitual sleep episode. Asterisks indicate significant pairwise Bonferroni-corrected comparisons [* *p*< 0.05: * *p* < 0.001]. ms = milliseconds; h = hours; lapses = RT > 500 ms.

In the PVT task, the mean RT [F(_18, 468.33_) = 21.80] and lapses [F(_18, 230.02_) = 14.89] showed significant variation across the 39 h of the CR (all *p*’s < 0.001), with the worst performance happening during the late night to morning hours (Figure 1). For the RT, the test sessions after 11.5 h of being awake and from 19.5 h of being awake onwards were significantly slower than other sessions (*p*’s < 0.05). The number of lapses of attention significantly increased in all sessions from 19.5 h awake onwards (all *p*’s < 0.05).

**Figure 2:**
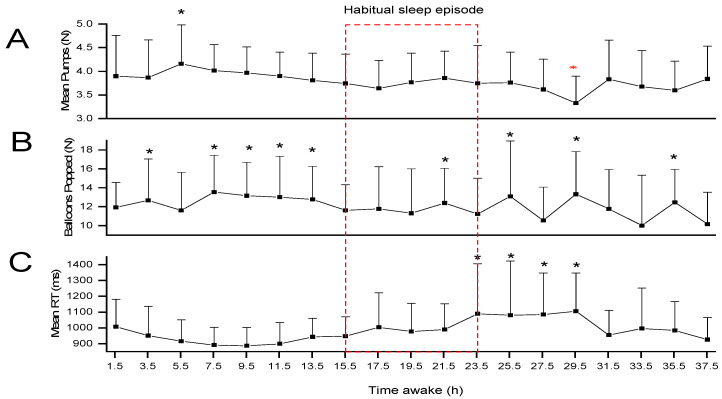
Mean (+standard error) pumps (**A**), balloons popped (**B**), and reaction time (**C**) on the balloon analogue risk task (BART) across the CR. The dashed red box indicates the timing of the habitual sleep episode. Asterisks indicate significant pairwise Bonferroni-corrected comparisons. [* *p* < 0.05: * *p* < 0.001]. N = number; ms = milliseconds; h = hours.

In the BART task, the number of mean pumps [F(_18, 233_) = 2.58, *p* < 0.001] and balloons popped [F(_18, 233_) = 2.29, *p* = 0.003] varied significantly across the CR. With respect to mean pumps, differences were found at 5.5 and 29.5 h awake (*p*’s < 0.05; Figure 2A and Figure S1). For the balloons popped, differences were found at multiple timepoints [3.5, 7.5, 9.5, 11.5, 13.5, 21.5, 25.5, 29.5, 35.5 h awake (all *p*’s < 0.05: Figure 2B and Figure S2)].

The RT performance on the BART task also showed significant variations across the CR [Mean RT F(_18, 346.06_) = 3.20, *p* < 0.001], with significantly slower mean RTs in the morning hours (23.5, 25.5, 27.5, and 29.5 h awake; all *p*’s < 0.05, Figure 2C and Figure S3). The mean pumping reward [F(_18, 232.98_) = 1.60, *p* = 0.06] and total amount collected [F(_18, 232.98_) = 1.59, *p* = 0.06] showed a similar trend (Figure S4).

### Additional Analysis

When comparing the BART tests carried out in the morning vs. the afternoon only, no significant differences were observed in the number of mean pumps, balloons popped, or total amount collected between the morning and afternoon hours (1.5 h awake vs. 7.5 h awake or 1.5 h awake vs. 9.5 h awake; all *p*’s > 0.05; see a summary of the results in Table S1).

## 3. Discussion

Results on the PVT revealed that under CR conditions, vigilance—as assessed using RTs and lapses of attention—remains relatively stable throughout the initial ~16 h, which corresponds to the habitual waking day. Vigilance then deteriorates during the habitual nighttime hours, reaching its worst in the morning just after the usual waketime. While vigilance subsequently improves over the late morning and into the afternoon, overall, it is worse after missing a night of sleep than during the same hours at the start of the CR. These results are in agreement with those previously reported, indicating an interaction between a circadian rhythm that promotes increased vigilance during the biological day and decreased vigilance during the biological night and a sleep–wake homeostatic process that degrades vigilance the longer one is awake [10,31,32,33].

In terms of the RT, the performance on the BART and the PVT followed similar trends, becoming significantly slower during the usual nighttime and slowing down even more after ~19.5 h awake, and then improving somewhat later in the day. However, the RT on the BART task was more stable across the CR than the RT was on the PVT. Why the RT on the decision-making task appears to be less sensitive to sleep loss than on the PVT is not clear. This could be because decision making involves a more complex process that goes beyond simply reacting to a stimulus. It requires deeper cognitive evaluations, such as assessing risks and benefits, considering relevant information, and weighing alternative options, any of which could interact with and potentially compensate for the attentional impairment.

Based on the reinforcement sensitivity theory, risk behavior is modulated by sensitivity to reward and punishment [34]. In the context of the present study, sensitivity to reward would be translated to the number of pumps (gains) and sensitivity to punishment to balloons popped (losses). Variations in performance across the CR relating to a greater propensity for risk (reflected in a high number of pumps) were only observed around midday (before any sleep loss), and performance variations indicating a lower risk propensity were observed after 29.5 h awake. These results are inconsistent with previous studies that reported a greater risk propensity after sleep loss [27,28]. Nevertheless, the methodological differences between these investigations are substantial, including differences in the sleep-loss protocols, the types of decision-making tasks, and the methods of assessing performance, rendering a comparison of the findings challenging.

In terms of risk propensity being understood as sensitivity to punishment, the number of balloons popped varied throughout the CR, but did not seem to follow a predictable trend. This could mean that it may not matter what time of day it is (no circadian variation in sensitivity to punishment/loss) or that there is little to no impact of sleep loss on sensitivity to punishment or loss. Alternatively, it may be that with sleep loss, the individual loses interest or has a sense of futility towards punishment or loss. Some studies have reported that when the amount of real incentive is increased, decision making and neural activity may change [35,36,37]. In this case, our results might be explained by the fact that the participants did not experience any real punishment or loss based on their task performance, and if instead they could have realized actual monetary rewards, then their task performance might have differed.

Finally, when we attempted to replicate the previously reported diurnal variations in risky decision making [23] by comparing non-sleep-deprived test sessions (morning vs. afternoon) on the BART, we did not find any significant differences between the morning and afternoon test performances. However, our sample (N = 13) was smaller than that of the previous study (where sample size was N = 28), and our post hoc power analysis indicated that it was insufficient to detect morning vs. afternoon differences in performance. However, our sample size was sufficient to find circadian effects in the reaction time performances across the CR for both PVT and BART outcomes.

It is also possible that we could not replicate the prior findings because our participants could not realize any actual financial reward related to their performance, whereas in the prior studies, the participants did receive real financial gains associated with their performance on the task.

There are some limitations in the current study. First, the sample was relatively small, which may have impacted our ability to detect small differences in performance. Because the study was not originally designed to detect diurnal variations in decision making, no *a priori* sample size calculations were conducted for this specific purpose.

Also, previous studies have shown that age is an important factor to consider when studying risky decision making [27], so the age range in our study, which was 21–31 years old, may be a limitation. As noted above, the reward condition on the BART was hypothetical, and prior research has demonstrated that real vs. hypothetical financial incentives may change how individuals behave. Therefore, our findings might have differed if the participants had a financial incentive when performing the task. Finally, we assessed risky decision making using only behavioral tasks. In the future, it may be beneficial to use multiple types of decision-making tasks or to combine behavioral tasks with neurophysiologic techniques, such as fMRI and EEG, to better probe the components of task performance in order to understand how they change regarding the circadian phase and sleep loss.

## 4. Materials and Methods

### 4.1. Participant Recruitment and Eligibility Criteria

Participants were recruited from the community using online notices and advertisements. They had to be free of any acute or chronic medical and psychological condition and to be taking no medication (excluding hormonal birth control). Eligibility was determined using their medical history, a physical examination, electrocardiogram, screening blood tests (complete blood count and comprehensive metabolic panel), urinalysis, psychological questionnaires (Minnesota Multiphasic Personality Inventory-2 [38], Beck Depression Inventory-II [39], Symptom Checklist-90 [40], and the State-Trait Anxiety Inventory [41]), and an interview with a clinical psychologist [42].

Participants had to report no sleep disorders or chronic sleep complaints (assessed with the Pittsburg Sleep Quality Index [43]), had to have a habitual sleep duration between 7 and 9 h per night, have no history of regular night work or rotating shift work, no recent travel across more than two time zones, and no self-reported daytime sleepiness on the Epworth Sleepiness Scale [44].

### 4.2. Study Protocol

Participants were asked to keep a regular sleep schedule of 9 h time-in-bed at home for at least two weeks prior to the day of admission to their 5-day study. Participants were studied individually in the Intensive Physiological Monitoring Unit of the Brigham and Women’s Hospital Center for Clinical Investigation and remained in their study room for the duration of their study. After admission in the afternoon of Day 1, each participant was oriented to the battery of neurobehavioral tests (~25 min). They took 4 practice test batteries on Day 1 to become familiar with the requirements of each test. After a 9 h scheduled sleep episode at their habitual time, the participant woke to a Baseline Day. During the Baseline Day, they took the test battery every 2 h for a total of 7 times. After a second 9 h scheduled sleep episode, upon awakening on Day 3 the participant began a ~39 h CR. This consisted of continuous wakefulness in a semi-recumbent posture in bed with a dim light (<15 lux) and with the nutritional intake being divided into identical hourly snacks [45]. Test batteries were given every two hours throughout the CR, starting 1.6 h after waking, for a total of 19 CR testing times. After the CR, the participant was scheduled for a 10 h recovery sleep, and they were discharged in the early afternoon on Day 5.

### 4.3. Test Battery

#### 4.3.1. Psychomotor Vigilance Task (PVT)

The PVT assesses the visual reaction time (RT) and was used as an objective measure of sustained vigilance [46]. In it, the participant was instructed to focus their gaze on a fixation point on a computer monitor and to respond as quickly as possible with a button press when a stimulus appeared on the screen. The inter-stimulus interval varied between 2 and 10 s, and the task was scheduled for 10 min, resulting in approximately 70–100 trials per test session. This task has been demonstrated to be sensitive to the circadian phase [47,48,49] and to both acute and chronic sleep loss [30,48], while not showing any long-lasting training effects.

#### 4.3.2. Balloon Analogue Risk Task (BART)

The BART is a validated assessment of risk-taking behavior [50]. In it, the participant is shown a balloon on the computer screen and instructed to either inflate the balloon or collect money. Each time the participant inflates the balloon, the monetary compensation increases, but there is also a higher possibility that the balloon will pop. The potential reward is lost if the balloon pops. The time of the task varied from 2–6 min per session, depending on how long the participant took to respond to a total of 30 balloons that were presented in each session. Each balloon had a different probability of popping that was arranged through constructing an array of N numbers. The number 1 was designated as indicating a balloon explosion. On each pump of the balloon, a number was selected without replacement from the array. The balloon exploded if the number 1 was selected. The maximal hypothetical reward was one dollar for each successful pump.

#### 4.3.3. Data Analysis

For the PVT, the mean RT and lapses of attention were used in the analyses. RTs > 500 milliseconds (ms) were considered lapses. Any RT < 100 ms was considered a false start and was excluded from subsequent analyses [46,51,52].

For the BART, the indexes used to assess performance were as follows: mean pump reward (the amount added to the reward after a balloon inflation that did not pop the balloon), total amount collected, and the mean RT (the time the participant took to decide to pump or collect each balloon). The mean pumps (mean number of pumps per balloon) and the balloons popped (number of balloons that popped) were considered measures of risk preference [50].

The RTs in both tasks did not follow a normal distribution (Kolmogorov–Smirnov *p*’s > 0.05), and we therefore applied a reciprocal transformation of the RTs on each task to better approximate a normal distribution.

Statistical analyses were performed using a mixed model regression analysis on raw data, incorporating TIME AWAKE as a fixed factor and PARTICIPANT as a random factor in the model. Due to the increased family-wise error of the 19 comparisons, a Bonferroni correction was applied.

To determine if there were learning or practice effects on the tasks, a variable CONDITION (baseline vs. CR) was included in the model as a fixed factor and the interaction CONDITION*TIME AWAKE was explored.

The package SPSS 28.0 software for Windows (IBM, Armonk, NY, USA) was used for all above analyses.

Finally, to compare our data with those previously reported [23] in which a significant time of day effect was observed for the average number of pumps and the total reward amount but not the balloons popped, we used paired Student’s t-tests on the average number of pumps, balloons popped, and total amount collected during two daytime tests. For methodological similarity, we compared a morning test (taken after ~1.5 h awake) to an afternoon test (tests at both 7.5 and 9.5 h awake) during the CR. For those comparisons, post hoc calculations on our observed data using G*Power 3.1.9.7 (Heinrich Heine University, Düsseldorf, Germany) indicated that a sample size of at least n = 40 would have been required to have 80% power at α = 0.05 to detect an effect size of −0.404 (Cohen’s d, as reported in [23]) between 1.5 and 7.5 h awake in mean pumps, and a sample size of n = 23 would have been required to have 80% power to detect an effect size of −0.543 between 1.5 and 7.5 h awake in the total reward amount. Similarly, a sample size of n = 40 and n = 23 would have been required to have 80% power at α = 0.05 to detect differences between 1.5 and 9.5 h awake in mean pumps and the total reward amount, respectively.

## 5. Conclusions

In the present study, the RT in a decision-making task was impaired during the morning hours after a night of sleep loss. Risky decision making related to seeking gains peaked during midday and was significantly inhibited by sleep loss after 29.5 h awake. The decision-making process related to loss avoidance followed an unstable pattern and did not vary significantly across the day or with increased sleep loss.

## Data Availability

The authors will make de-identified data from the current study available upon written request. Execution of a Materials Transfer Agreement is required if the data will be used in research supported by a for-profit company, per Mass General Brigham Institutional Review Board policy. The specific data to be shared will be PVT and BART related data with the associated wake duration information from the 7 baseline and 19 CR sessions.

## Supplementary Materials

The following supporting information can be downloaded at https://www.mdpi.com/article/10.3390/clockssleep6020020/s1, Figure S1. Mean Pumps (n) of the Balloon Analogue Risk Task (BART) across 39 h awake for each participant, Figure S2. Balloons popped (n) of the Balloon Analogue Risk Task (BART) across 39 h awake for each participant, Figure S3. Reaction time (ms) of the Balloon Analogue Risk Task (BART) across 39 h awake for each participant, Figure S4. Total Collected (A) and Mean Pumping Reward (B) (+ standard error, shown only in the positive direction) on the Balloon Analogue Risk Task (BART) across 39 h awake, Table S1. Comparison of day vs. evening data on Number of Pumps and Total Gains with results of Li et al. (2020) [23].
