# Supplementary material for: Time of Day and Sleep Deprivation Effects on Risky Decision Making
Source: Clocks Sleep. 2024 Jun 20;6(2):281–90. doi: 10.3390/clockssleep6020020 (PMC11202614; doi:10.3390/clockssleep6020020)

clocks & sleep

MDPI

Table S1. Comparison of day vs. evening data on Number of Pumps and Total Gains with results of Li et al., (2020).

	Li et al., (2020) N = 28			Present study N = 13				
	**Morning M(SD)**	**Afternoon M(SD)**	**T; p values**	**Morning M(SD)**	**A1 M (SD)**	**A2 M (SD)**	**T; p values A1**	**T; p values A2**
Mean of pumps	6.06(1.16)	6.42 (1.41)	-2.135**;** **0.04**	3.09(0.9)	4 (0.5)	4 (0.5)	-0.12; 0.55	−0.08; 0.93
Balloons popped	17.36(3.73)	16.89 (4.6)	0.762; 0.45	11.92(2.63)	16.89 (4.62)	13.15 (3.53)	-1.80; 0.09	-1.70; 0.10
Total gains	164(18.1)	174.29 (13.85)	−2.871**; **0.008**	80.4(13.4)	79.1 (12.4)	81.1 (12)	0.2; 0.8	−0.1; 0.9

Note. A1 = afternoon 1, 7.5 hours since wake; A2 = afternoon 2, 9.5 hours since wake time. Morning values are taken in the first session (1.6 hours since wake time)

Figure S1. Mean Pumps (n) of the Balloon Analogue Risk Task (BART) across 39 h awake for each participant.

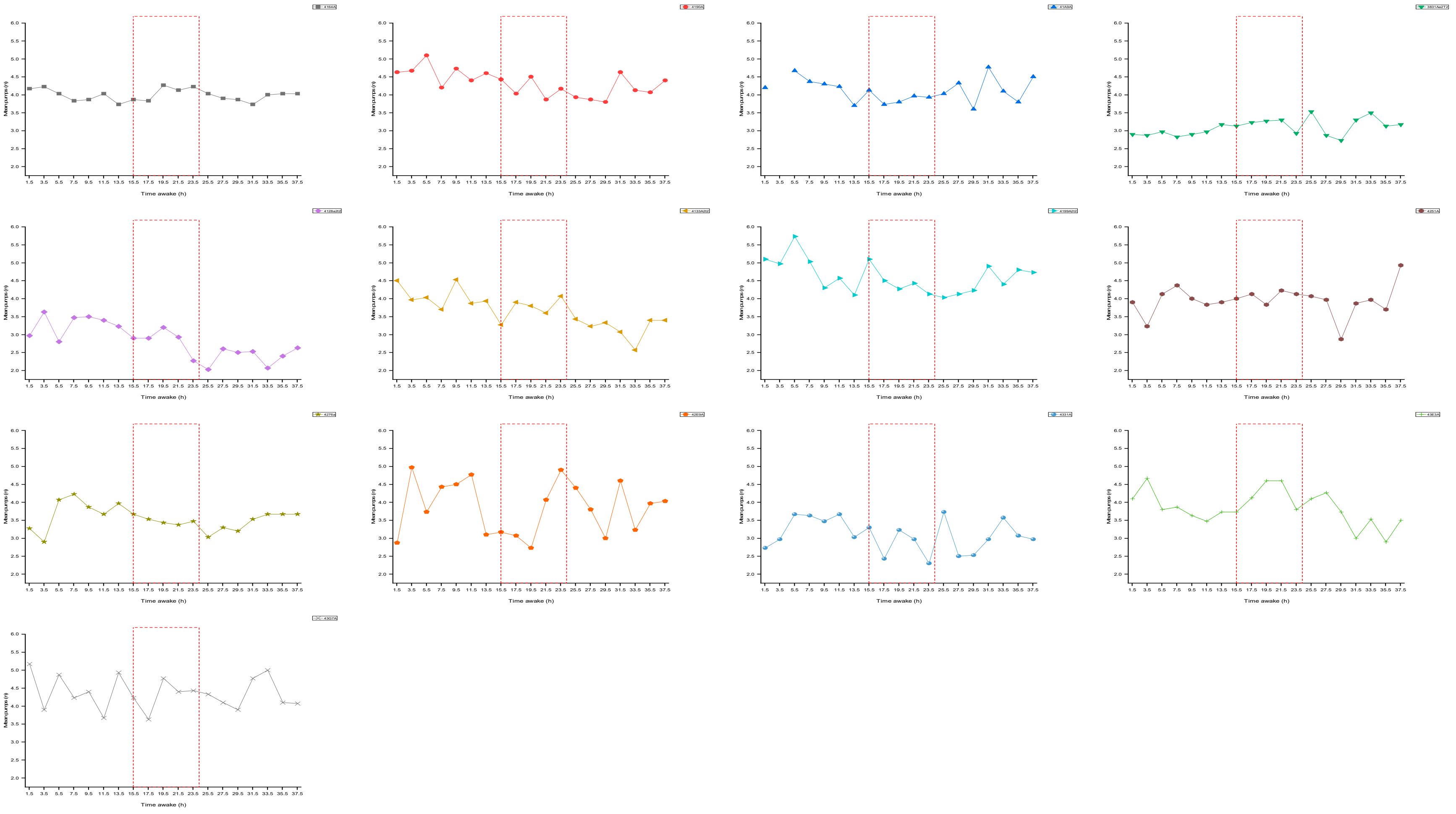

Figure S2. Balloons popped (n) of the Balloon Analogue Risk Task (BART) across 39 h awake for each participant.

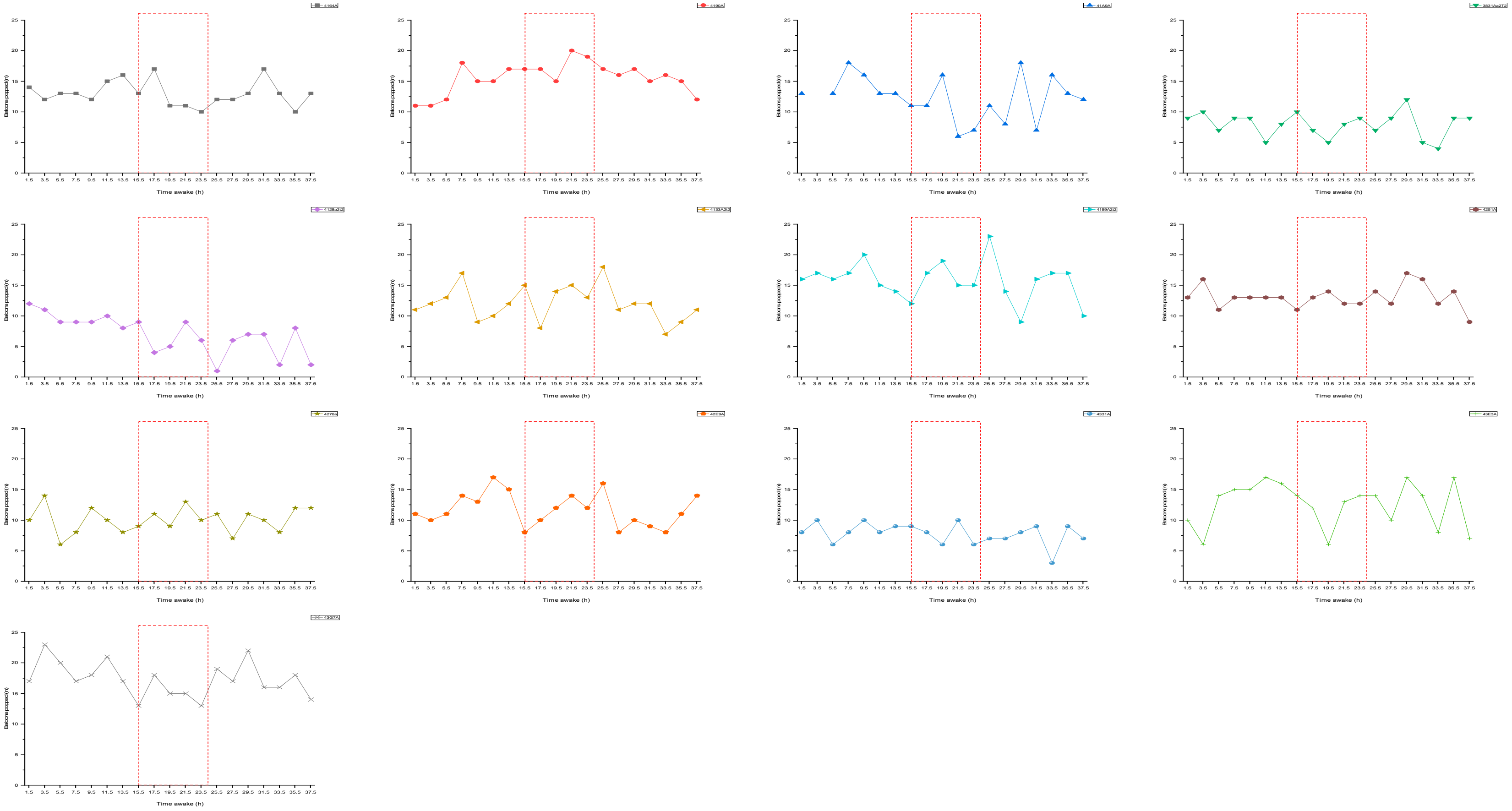

Figure S3. Reaction time (ms) of the Balloon Analogue Risk Task (BART) across 39 h awake for each participant.

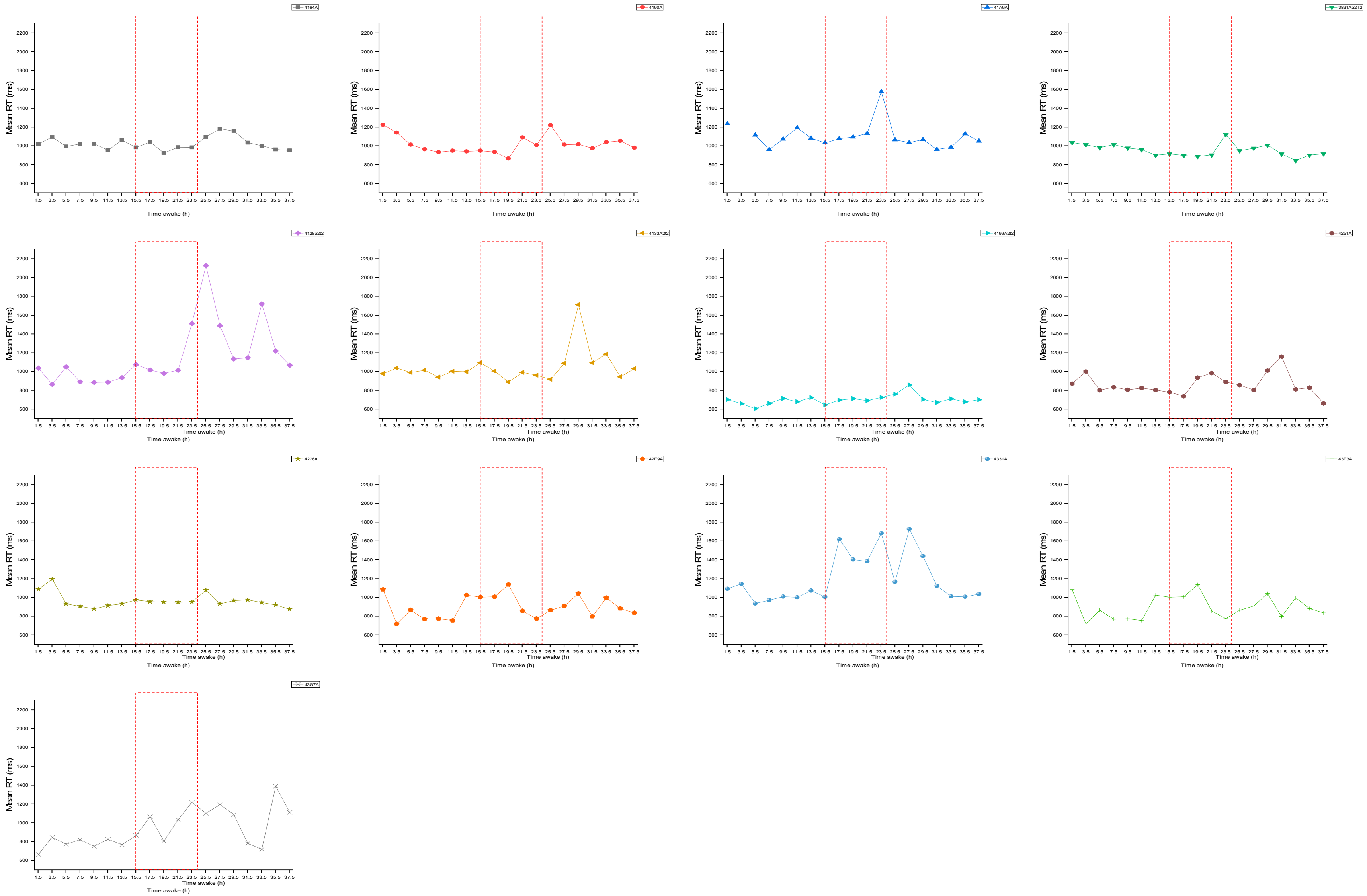

Figure S4. Total Collected (A) and Mean Pumping Reward (B) (+ standard error, showed only in plus direction) of the Balloon Analogue Risk Task (BART) across 39 h awake. Asterisks indicate significant pairwise Bonferroni-corrected comparisons.

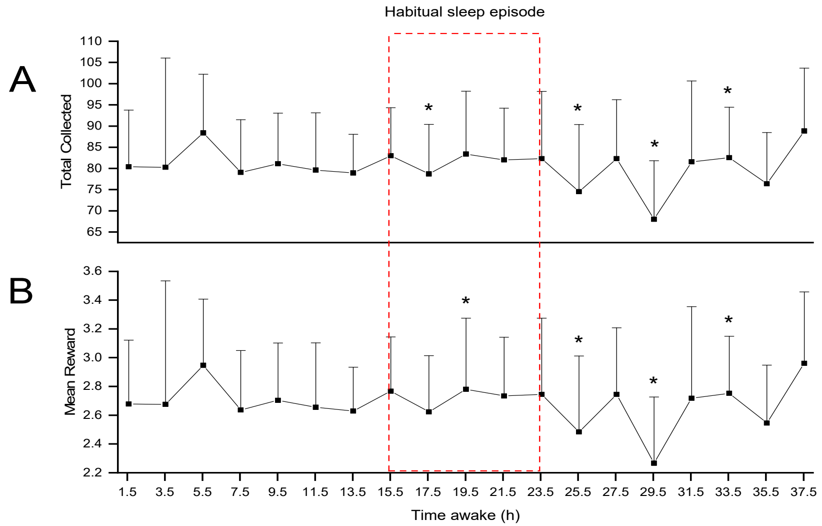

Supplement: Supplementary file 1 [file clockssleep-06-00020-s001.zip › clockssleep-3005165-supplementary.pdf]
